# Supplementary figures and images for: Host 5-HT affects Plasmodium transmission in mosquitoes via modulating mosquito mitochondrial homeostasis
Source: PLoS Pathog. 2024 Oct 15;20(10):e1012638. doi: 10.1371/journal.ppat.1012638 (PMC11508672; doi:10.1371/journal.ppat.1012638)

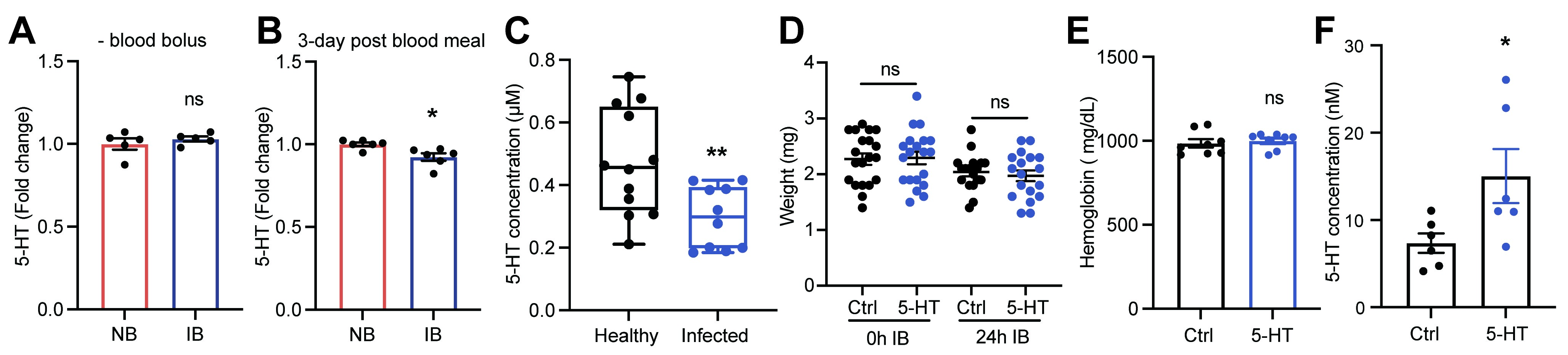

Supplement: S1 Fig — (A) Fold change of 5-HT levels in the mosquito midguts 24 h post normal blood (NB, n = 5) and P. berghei infected blood (IB, n = 5) analyzed by ELISA. The blood bolus was removed from the midgut 24h post blood meal. Sixty midguts were pooled for one sample. Each dot represented one biological replicate. Data were pooled from two independent experiments and shown as mean ± SEM. (B) Fold change of 5-HT levels in the mosquito midguts 3 days post normal blood (NB, n = 6) and P. berghei containing infectious blood (IB, n = 6) analyzed by ELISA. Results represented that the blood was digested completely in the midgut 3 days post blood meal. Thirty midguts were pooled for one sample. Each dot represented one biological replicate. Data were pooled from two independent experiments and shown as mean ± SEM. (C) 5-HT concentrations in the sera of healthy (Healthy, n = 12) and Plasmodium infected adults (Infected, n = 10). Each dot represented an individual and the data were shown as mean ± SEM. (D) The weight of control and 1 μM 5-HT treated mosquitoes 0 h (Ctrl, n = 20, 5-HT, n = 20) and 24 h (Ctrl, n = 18, 5-HT, n = 18) post- infection. Each dot represented an individual mosquito. Data were pooled from two independent experiments and shown as mean ± SEM. (E) Hemoglobin concentrations in the control (Ctrl, n = 8) and 1 μM 5-HT (5-HT, n = 8) treated mosquitoes (n = 6) 0 h post-infection. Each dot represented 30 mosquito midguts and the data were shown as mean ± SEM. (F) 5-HT concentrations in the control (n = 6) and 1 μM 5-HT treated mosquitoes (n = 6) 4 days post 5-HT treatment (24 h prior to blood feeding). Twenty-five mosquitoes were pooled for one sample. Each dot represented one biological replicate. Data were pooled from two independent biological experiments and shown as mean ± SEM. Significance was determined by two-sided Student’s t test. *p < 0.05, **p < 0.01, ns, not significant. (TIF) [file ppat.1012638.s001.tif]

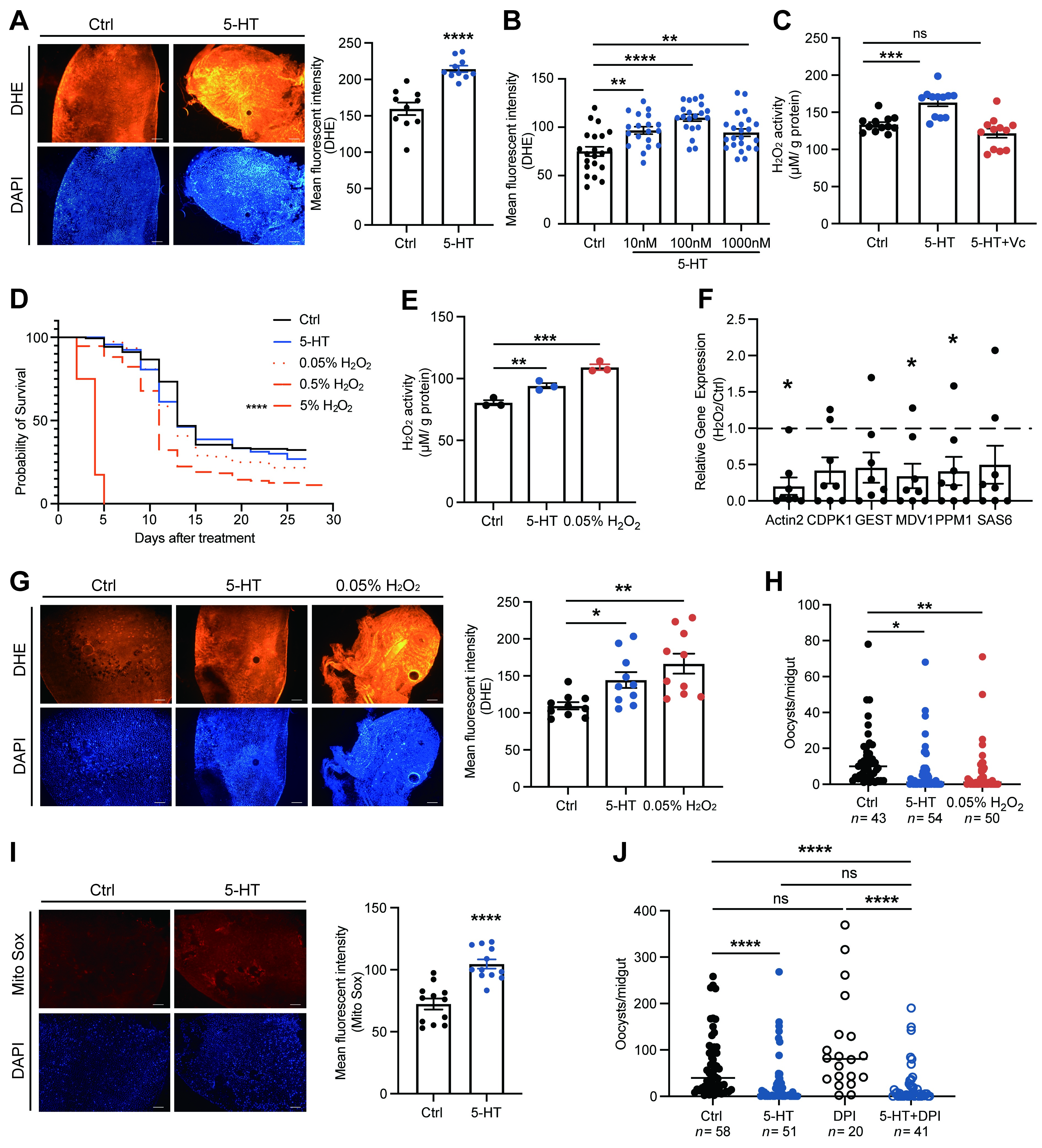

Supplement: S2 Fig — (A) DHE staining (red) in the midguts of control and 5-HT (1 μM) treated mosquitoes 15 min post-infection. Nuclei were stained with DAPI (blue). Representative images were shown (left). Mean fluorescent intensity was measured and calculated (right). Each dot represented an individual mosquito midgut. Data were pooled from two independent experiments and shown as mean ± SEM. Scale bar, 25 μm. (B) Quantification of fluorescent DHE intensity in control and 10 nM-, 100 nM- and 1000 nM-5-HT treated mosquitoes 24 h post-infection. Each dot represented an individual mosquito. Data were pooled from two independent experiments and shown as mean ± SEM. (C) The levels of H2O2 in the midguts of control, 5-HT and 5-HT + Vc treated mosquitoes 24 h post-infection. Each dot represented an individual mosquito. Data were pooled from three independent experiments and shown as mean ± SEM. (D) Survival assay of mosquitoes treated with 5-HT and different concentrations of H2O2 (n = 40–159 mosquitoes per group). Results were pooled from two independent experiments. (E) The levels of H2O2 in the midguts of control, 5-HT- (1 μM) and 0.05% H2O2- treated mosquitoes 24 h post-infection. Each dot represented an individual mosquito. Data were pooled from three independent experiments and shown as mean ± SEM. (F) Fold changes of male gametogenesis associated genes in the midguts of control (n = 8) and 0.05% H2O2-treated (n = 8) mosquitoes 15 min post-infection. The expression level of the target gene was normalized to S7. The relative expression level of target genes in 0.05% H2O2-treated mosquitoes was normalized to that in controls. Each dot represented five mosquito midguts. Data were shown as mean ± SEM. (G) DHE staining (red) in the midguts of control, 5-HT- (1 μM) and 0.05% H2O2- treated mosquitoes 15 min post-infection. Nuclei were stained with DAPI (blue). Representative images were shown (left). Mean fluorescent intensity was measured and calculated (right). Each dot represented an indiv [file ppat.1012638.s002.tif]

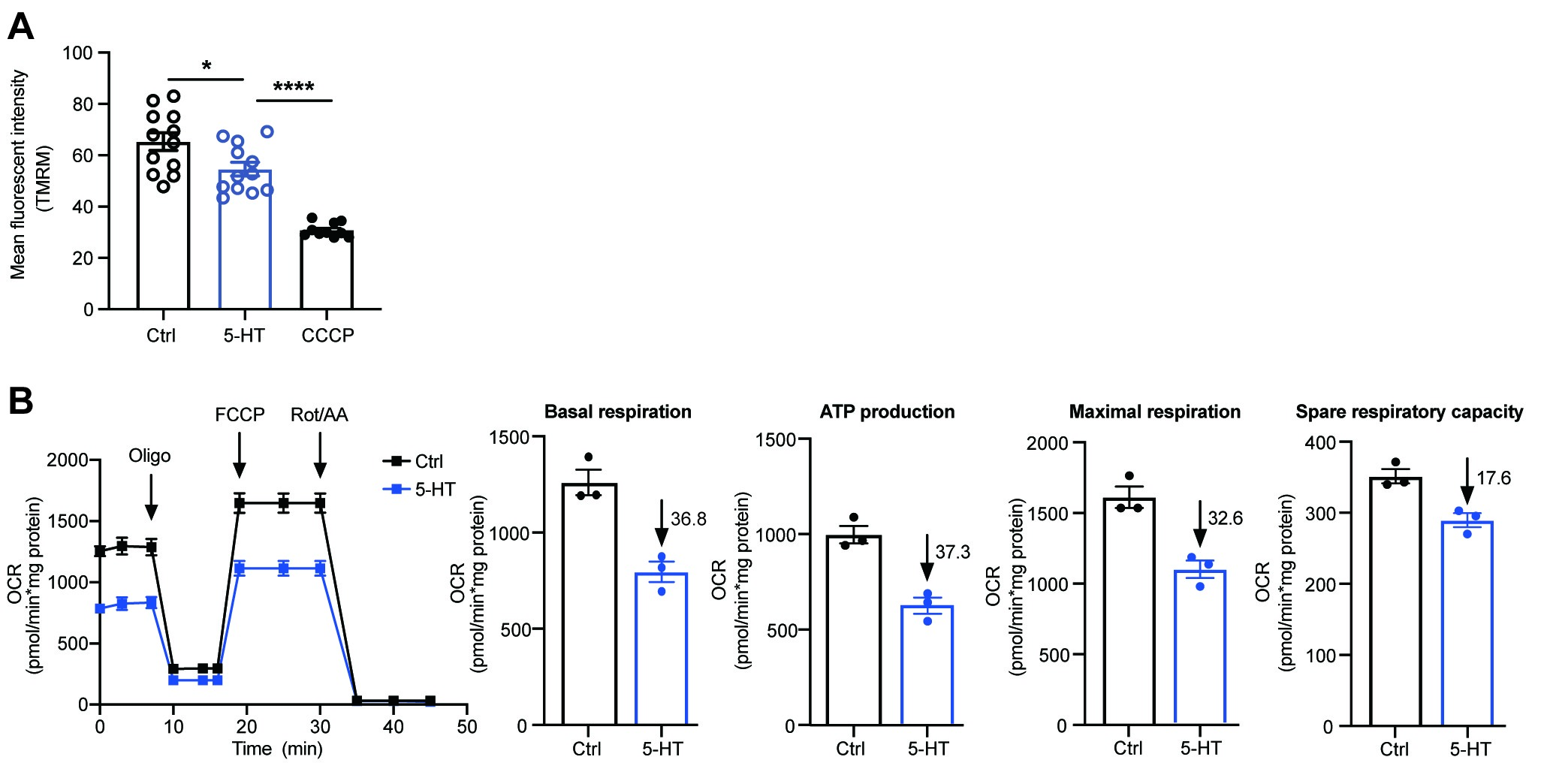

Supplement: S3 Fig — (A) The quantification of mitochondrial membrane potential of MSQ43 cells treated with 5-HT and CCCP were analyzed by TMRM staining. Each dot represented an independent experiment and the data were shown as mean ± SEM. (B) Oxygen consumption rate (OCR) in control and 5-HT treated MSQ43 cells. Results were pooled from three independent experiments. Each dot represented an independent experiment and data were shown as mean ± SEM. Significance was determined by ANOVA with Tukey’s test in (A) and two-sided Student’s t test in (B). *p < 0.05, ****p < 0.0001. (TIF) [file ppat.1012638.s003.tif]

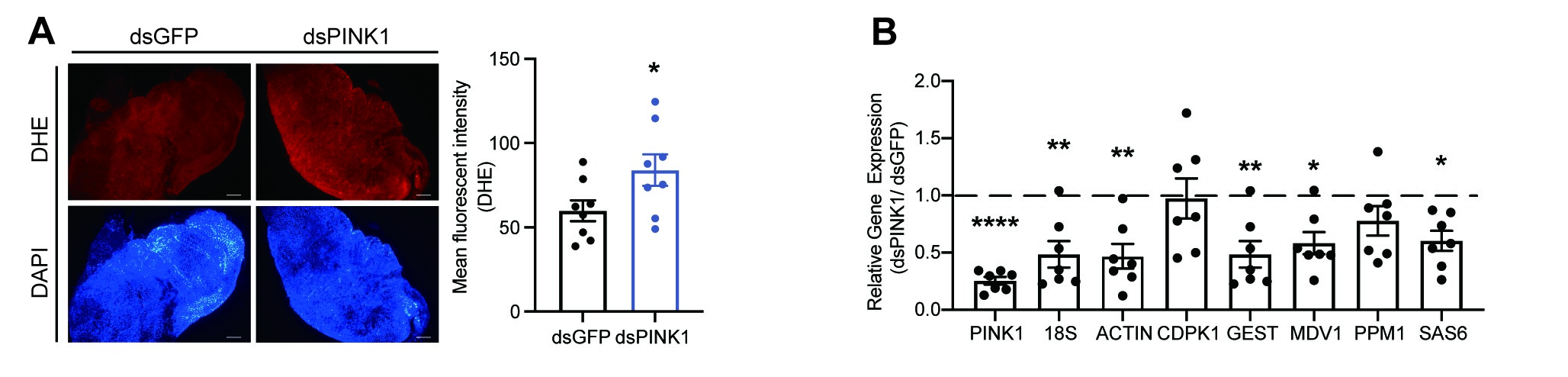

Supplement: S4 Fig — (A) DHE staining (red) in the midguts of dsGFP and dsPINK1 treated mosquitoes 15 min post-infection. Nuclei were stained with DAPI (blue). Representative images were shown (left). Mean fluorescent intensity was measured and calculated (right). Each dot represented an individual mosquito midgut. Data were pooled from three independent experiments and shown as mean ± SEM. Scale bar, 25 μm. (B) Fold changes of male gametogenesis associated genes in the midguts of dsGFP (n = 8) and dsPINK1 (n = 8) treated mosquitoes 15 min post-infection. The expression level of the target gene was normalized to S7. The relative expression levels of target genes in dsPINK1 treated mosquitoes were normalized to those in controls. Each dot represented five mosquito midguts. Data were shown as mean ± SEM. Significance was determined by two-sided Student’s t test in (A) and (B). *p < 0.05, **p < 0.01, ****p < 0.0001. (TIF) [file ppat.1012638.s004.tif]

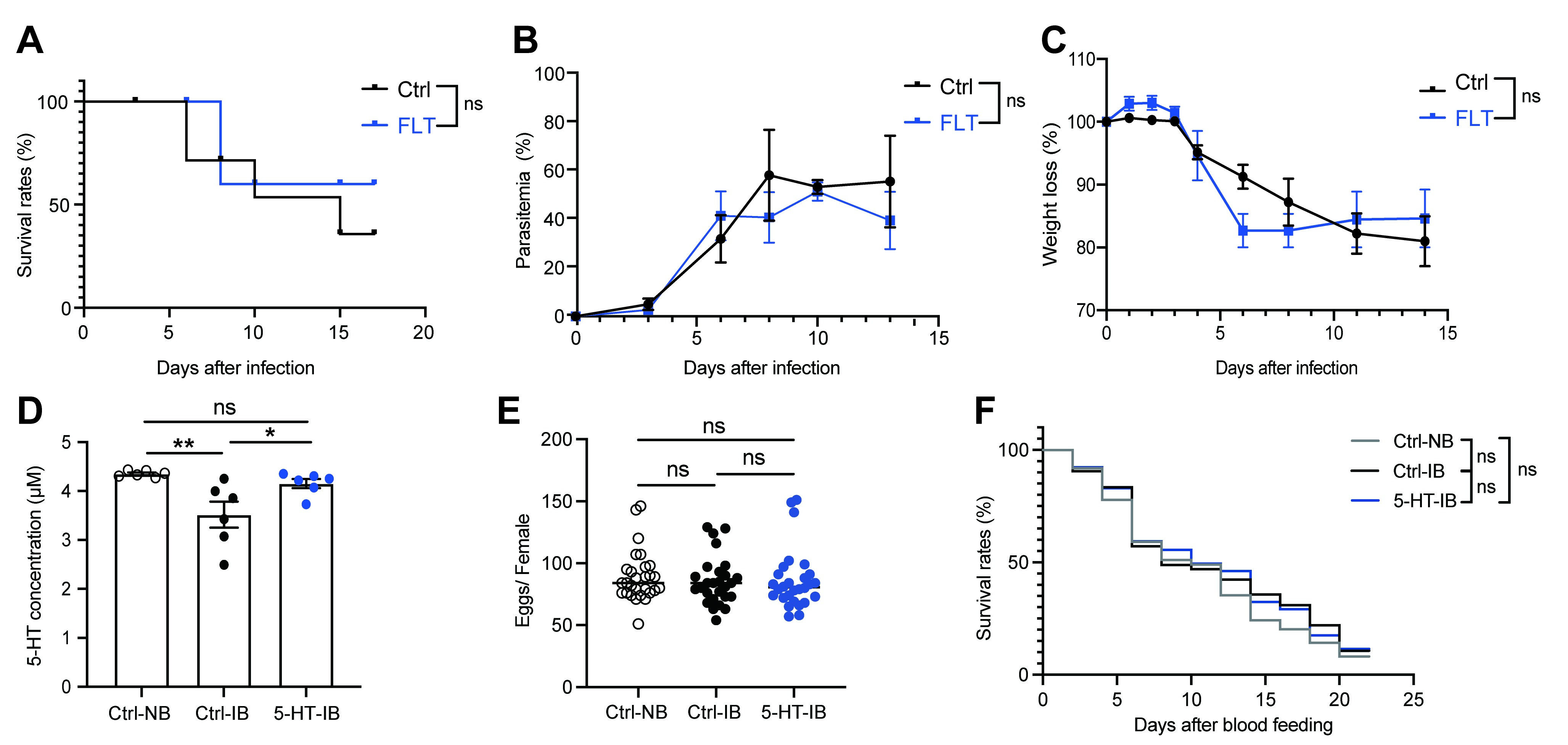

Supplement: S5 Fig — (A-C) Survival (A, n = 8 mice per group), Parasitemia (B, n = 5 mice per group) and Weight loss (C, n = 5 mice per group) of mice following saline and fluoxetine treatment. (D) 5-HT concentrations in the midguts of mosquitoes fed on non-infected (Ctrl-NB, n = 6), Plasmodium infected (Ctrl-IB, n = 6) and Plasmodium infected + 5-HT injected (5-HT-IB, n = 6) mice 24 h post-infection. Thirty midguts were pooled for one biological sample. Each dot represented one biological replicate. Data were pooled from two independent experiments and shown as mean ± SEM. (E) Reproductivity of female mosquitoes fed on non-infected (Ctrl-NB), Plasmodium infected (Ctrl-IB) and Plasmodium infected + 5-HT injected (5-HT-IB) mice 72 h post-infection. Each dot represents an individual mosquito. Data were pooled from two independent experiments and horizontal lines represented the medians. (F) Survival rate of mosquitoes following fed on non-infected (Ctrl-NB), Plasmodium infected (Ctrl-IB) and Plasmodium infected + 5-HT injected (5-HT-IB) mice blood. Significance was determined by A Log-rank (Mantel-Cox) test in (A) and (F), two-sided Student’s t test in (B) and (C), and ANOVA with Tukey’s test in (D) and (E). *p < 0.05, **p < 0.01, ns, not significant. (TIF) [file ppat.1012638.s005.tif]
